# Rare Complications Following Laparoscopic Sleeve Gastrectomy

**DOI:** 10.3390/jcm13154456

**Published:** 2024-07-30

**Authors:** Amanda Belluzzi, Jack W. Sample, Katie Marrero, Daniel Tomey, Suraj Puvvadi, Ishna Sharma, Omar M. Ghanem

**Affiliations:** 1 Department of Surgery, Mayo Clinic, Rochester, MN 55095, USA; amandabelluzzi@gmail.com (A.B.); sample.jack@mayo.edu (J.W.S.); 2 Department of Surgery, Rovigo Hospital, 45100 Rovigo, Italy; 3 Carle Foundation Hospital General Surgery Residency, Champaign, IL 61801, USA; 4 Department of General Surgery, Houston Methodist Hospital, Houston, TX 77030, USA; daniel_tomey93@hotmail.com; 5 College of Health Solutions, Arizona State University, Phoenix, AZ 85004, USA; 6 St. Peter’s Health Partners Bariatric and Metabolic Care, Albany, NY 12208, USA; ishna.sharma@gmail.com; 7 Division of Metabolic and Abdominal Wall Reconstructive Surgery, Mayo Clinic, Rochester, MN 55095, USA

**Keywords:** rare complications, sleeve gastrectomy, bariatric surgery

## Abstract

Metabolic and bariatric surgery (MBS) is the most effective and durable therapeutic intervention for patients with obesity. In recent years, laparoscopic sleeve gastrectomy (SG) has become the most commonly performed primary MBS procedure owing to its technical feasibility and excellent short-term outcomes. Despite these favorable results and perceived advantages, SG is associated with several unique complications. Complications such as a postoperative leak or bleeding have been more commonly observed and reported than others, and their management approaches are well described. However, other complications following SG are far less familiar to surgeons, which may delay recognition and result in poor patient outcomes. Of these complications, we describe splenic injuries; esophageal perforation; staple line malformations; stapling of intraluminal devices; phytobezoar formation; gastro-colic, gastro-pleural and gastro-bronchial fistula; pancreatic leak; and portomesenteric venous thrombosis. It is paramount for surgeons to be aware of these underreported issues and have the resources to learn how to recognize and manage them when they arise. This review aims to describe rare (i.e., reported incidence <1%) and underdescribed complications after SG, focusing on causes, clinical presentation, prevention strategies, and management.

## 1. Introduction

The prevalence of obesity, defined by a body mass index (BMI) over 30 kg/m^2^, is a worldwide health concern affecting nearly 35% of the adult population in the United States [1,2,3,4]. People with obesity are at increased risk for numerous obesity-related comorbidities including type 2 diabetes mellitus (T2DM), cardiovascular disease, and cancer [2,3]. Economically, it is estimated that obesity and its associated comorbidities account for 17.8% of total healthcare expenditures, amounting to 2.42% of the total gross domestic product (GDP) [2,3].

Over the years, metabolic and bariatric surgery (MBS) has made great strides in addressing the global prevalence of obesity. Among all MBS procedures, laparoscopic sleeve gastrectomy (SG) is the most commonly performed surgical treatment option for patients with obesity, accounting for 61% of all bariatric procedures performed. Laparoscopic SG is considered to be a technically feasible and safe surgery with low complication and mortality rates [4,5,6,7,8]. Among the most commonly reported complications, gastric leak is a dreadful complication following laparoscopic LSG, reported in around 0.16% of the cases according to the metabolic and bariatric surgery accreditation and quality improvement program (MBSAQIP). Nevertheless, according to IFSO, the overall incidence of postoperative complications for LSG is 2.12% [4,5,6,7]. Other commonly described postoperative complications include hemorrhage and gastric stenosis. SG has demonstrated promising outcomes with a 5-year excess body weight loss of 60% and an improvement or complete resolution of comorbidities while maintaining a low morbidity rate [4,5,6,7,8]. Despite these excellent outcomes, complications following bariatric procedures are inevitable. Common intraoperative complications of SG include bleeding, which can occur during the division/takedown of greater curvature vessels or during gastric stapling. Additionally, splenic infarct or ischemia may result during the division of the most proximal vascular fundus attachments [6]. There are several notable postoperative complications after SG that may result in undue patient burden and, in some cases, require a revisional procedure. Common early postoperative complications, defined as those within the first 30 days following a procedure, include hemorrhage, staple line leak, and abscess formation, while common late postoperative complications include gastric stenosis, nutritional deficiencies, and gastroesophageal reflux diseases [6,9].

With the popularity and growing prevalence of SG procedures, there is a growing need for the review and description of the less common complications. While numerous studies have discussed common complications associated with SG, there is a paucity of literature addressing the range of rare issues after SG. The reason why these are less reported is because of how rare they are, and most of the literature focuses on the more common complications. Having the knowledge of postoperative complications that may occur after SG, particularly rare ones, surgeons may be better equipped to recognize these problems earlier and improve patient outcomes. This review aims at describing rare intraoperative and postoperative complications of sleeve gastrectomy while addressing their causes, prevention, and management.

## 2. Sleeve Gastrectomy Surgical Procedure

The laparoscopic SG procedure encompasses the mobilization of the greater curvature of the stomach by the division of greater curvature vessels starting 4 cm away from the pylorus up to the gastric fundus, at the level of short gastric vessels (SGV). A 40-French tube is inserted in the stomach, around which the sleeve is fashioned.

## 3. Splenic Complications

Sleeve gastrectomy (SG) is a widely performed procedure for obesity but carries risks such as intraoperative splenic bleeding. Due to the spleen’s close proximity and attachments to the greater curvature of the stomach, splenic injury is a potential intraoperative complication encountered during SG. While uncommon, splenic injury may lead to significant patient morbidity and sometimes necessitates splenectomy [10]. The spleen’s proximity to the surgical field and its extensive vascular supply makes it vulnerable to injury [11,12]. Even minor trauma can cause significant hemorrhage, requiring immediate intervention. In order to minimize and prevent splenic injury during SG, it is paramount to avoid excessive manipulation or unnecessary traction on perisplenic peritoneal folds and reach the best and most adequate exposure and visualization of all anatomic structures. Another technical aspect for minimizing the risk of splenic injury may include the division of the SGV as close as possible to the stomach to avoid the risk of a splenic branch damage. The incidence of splenic injury requiring splenectomy during laparoscopic sleeve gastrectomy is as low as 0.1% [13]. Such injuries typically occur due to traction or laceration during the mobilization of the gastric fundus. Management ranges from conservative measures like direct pressure to splenectomy, depending on the severity of the injury [10]. Early recognition and management are crucial for reducing morbidity [13,14]. However, if bleeding is difficult to control, splenectomy might be indicated.

Splenic infarction, although rare during gastric sleeve surgery, results from the interruption of the splenic blood vessels. Owing to the spleen’s ability to tolerate ischemia, splenic infarction is most often asymptomatic and incidentally discovered on postoperative imaging [15,16,17]. If symptoms are present, they manifest as left upper quadrant pain. Analgesia is the primary approach for symptomatic cases, typically involving NSAIDs or acetaminophen [18]. Most patients recover fully without surgical intervention, emphasizing the efficacy of conservative treatment. In order to reduce the risk of splenic infarction, it is important to practice preventative measures during surgery, such as avoiding excessive traction and maintaining hydration.

Splenic abscess is another rare and potentially life-threatening complication after SG, with a reported incidence of 0.14 to 0.7% after abdominal surgery as suggested in autopsy series [19]. Few cases of splenic abscess after SG have been described in the literature [19,20]. According to a recent systematic review from Buksh et al., 23 cases (85.2%) of splenic abscess were described after primary SG [21]. The most common etiologies and risk factors for the formation of a splenic abscess are the following: bacterial translocation or seeding from other infection sites, patient immunosuppression, hematologic disorders, splenic trauma leading to splenic infarction, intravenous drug addiction with septic emboli to the spleen, pancreatitis pancreatic abscess, pancreatic adenocarcinoma, gastrointestinal perforation and peritonitis (especially colonic) [18,19,20,21,22]. Sakran et al. reported that in most cases of splenic abscess after SG, the patient presents within 98.6 ± 132.7 days after the primary operation [19]. Patients may present with fever, left upper quadrant abdominal pain, nausea, and vomiting. In most cases, symptoms are non-specific, making the diagnosis challenging. The diagnostic process mainly relies on the combination of clinical presentation, blood tests (namely increased WBC count, CRP, and procalcitonin), and imaging. According to the literature, a contrast-enhanced abdominal CT scan remains the gold standard in the diagnosis since it can provide paramount information regarding the main characteristics of the splenic abscess (unilocular versus multilocular, presence of inner air component), which may have an impact on treatment choice [19,20,21,22]. The gold standard treatment for splenic abscess after SG is still under debate. The initial nonoperative approach includes intravenous broad-spectrum antibiotic therapy and percutaneous drainage without splenectomy; aspiration rather than drainage can be an option in cases of unilocular abscess less than 5 cm in size [23,24,25,26,27]. In cases of persisting symptoms or multilocular abscesses, a splenectomy may be required [21,22,28,29]. Splenectomy should be reserved for patients with complex abscess features, which may include multilocular composition and multiple or recurrent splenic abscesses where percutaneous drainage has failed. Additionally, asplenia is associated with post-splenectomy vaccine administration protocols, not to mention the impaired immunity status reported in the bariatric population compared to the non-bariatric one [30,31].

In conclusion, splenic injury during gastric sleeve surgery is uncommon but can be a cause of patient morbidity. Intraoperative bleeding, infarction, and abscess formation require prompt recognition and appropriate management. Conservative treatments are often effective, but splenectomy remains necessary in certain cases to prevent severe complications. Adherence to care and meticulous surgical techniques are vital in reducing the incidence of these complications and improving patient care. Careful surgical techniques, including gentle spleen handling and ensuring adequate perfusion, help minimize the risk of abscess formation. Early recognition and management are paramount for improving patient outcomes.

## 4. Esophageal Perforation

Esophageal injury is a serious clinical condition associated with high morbidity and mortality [32]. Even with early recognition and aggressive treatment, full-thickness esophageal perforation can quickly lead to rapidly developing sepsis and, in extreme cases, patient demise. Although emerging medical technologies are beginning to offer new treatment alternatives for these injuries, the general management principles for these injuries includes early detection, aggressive medical resuscitation, and surgical localization of the injury with debridement, drainage, and consideration of esophageal diversion.

Esophageal perforation is an exceedingly rare complication of sleeve gastrectomy (SG), an operation that typically does not directly involve surgical manipulation of the esophagus. However, iatrogenic esophageal injuries may occur secondary to the passing of orogastric calibration devices that are routinely used during this procedure [33,34,35]. Although the use of such orogastric devices during SG is seemingly benign, calibration tube-induced esophageal injury may be an overlooked source of serious patient morbidity following bariatric surgery. While reports of this mechanism of injury are limited in sleeve cases, several studies have discussed the incidence of orogastric tube injury during foregut surgery [35,36]. A review by Zhang et al. identified two cases of esophageal perforation due to bougie placement in a review of 1223 foregut surgeries [36]. Several recent case reports have described events of esophageal perforation that occurred secondary to bougie advancement during SG [37,38,39,40,41]. In a retrospective review of 390 patients who underwent bariatric surgery at a single institution, Aljehani et al. identified three instances of thoracic esophageal perforation caused by bougie advancement during SG [42]. According to the work of Gagner et al., the use of the ViSiGi 3DTM (Boehringer Labs, LLC, Phoenixville, PA, USA), a novel calibration system employing a safe level of suction and performing all functions with one insertion, may reduce the risk of perforation. Secondary to its fenestration pattern, the distal tip is more flexible, which may have perceived advantages [40,43]; however, these suggestions are yet to be validated. Among different methods of calibration, in some cases, the endoscope can be safer than the use of the bougie its self. Moreover, endoscopic calibration was reported to be associated with lower postoperative complications (i.e., gastric leak, esophageal perforation and postoperative digestive bleeding) [37,40,41,42,43]. However, there is no level 1 evidence comparing calibration tubes. and surgeons utilize tubes according to preference and availability. Nevertheless, it is paramount to utilize effective communication between multidisciplinary team members during calibration tube placement to ensure that these devices are safely passed in a careful and controlled fashion.

The esophagus has three areas of anatomic narrowing: the cervical esophageal constriction occurs at the cricopharyngeal sphincter, the thoracic esophageal constriction occurs where the aortic arch compresses against the posterior esophagus, and the abdominal esophageal constriction occurs where the esophagus enters the diaphragm forming the physiologic lower esophageal sphincter [44,45]. These anatomic narrowings, in addition to lacking a serosal layer, make the esophagus more susceptible to perforation. Iatrogenic perforations tend to occur either proximally near the hypopharynx or in the distal esophagus [44]. Bougie-related esophageal perforations during SG seem to follow this anatomic distribution, occurring primarily in the cervical esophagus [41,46] and distal esophagus [38,39,40,41] amongst the limited available reports. Esophageal perforations can be associated with the development of perforation-related sequelae, such as fistula formation. Full-thickness perforation of the esophagus may lead to an abnormal connection between the esophagus and surrounding airway structures (esophagopulmonary fistula) or develop a connection through the diaphragm to nearby abdominal structures. Draeger and colleagues report an unusual case of a patient who experienced an esophagopulmonary-splenopancreatic fistula following iatrogenic esophageal perforation after a SG [47].

Approaches to the management of esophageal injury continue to evolve as less invasive treatment modalities become increasingly sophisticated. However, the mainstay principles of management are well-established and include prompt recognition, meticulous patient monitoring, and optimized medical care with fluids, broad-spectrum antimicrobials, intravenous proton pump inhibitors, and nil per os. If identified intraoperatively, proper surgical attention is required to identify and repair the full extent of the injury. If identified postoperatively, these patients will likely require definitive management with antibiotics, drainage, and either endoscopic stent placement or surgical repair. The decision to utilize endoscopic versus surgical management is an area of debate. Stent placement is generally reserved for patients who are hemodynamically stable and is often dependent on factors such as location of injury, institutional access to endoscopy, and provider expertise. Despite advances in endoscopic management, surgery is often still required to avoid patient morbidity and mortality. The surgical approach is dependent on the location and extent of the injury and typically involves debridement of devitalized tissue, primary repair in layers, consideration of a vascularized pedicled flap, and placement of drains. In severe cases, temporizing procedures such as esophageal diversion may be necessary to allow for the patient to be adequately resuscitated before definitive repair is attempted. Despite early recognition and initiation of standard-of-care treatment, the mortality of these injuries remains high [33,39].

In conclusion, esophageal perforation is a rare but potentially life-threatening complication of sleeve gastrectomy that may occur during bougie advancement. Although uncommon, surgeons need to be aware of this complication given the criticality of early recognition and proper management, which often includes surgical repair. Extreme care should be used when placing and advancing calibration tubes during a sleeve gastrectomy.

## 5. Staple Line Malformation

**Figure 1:**
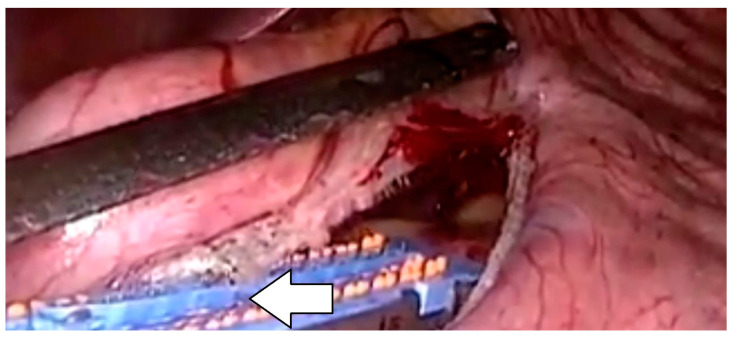
Staple line malformation.

Surgical stapler development revolutionized intestinal surgery and significantly progressed the field of metabolic and bariatric surgery. Staple line malformations during sleeve gastrectomy surgery, though rare, represent a significant complication with potential implications for patient outcomes [48]. According to the work of Makanyengo, the incidence of primary stapler malfunction ranged from 0.022% to 2.3% based on data collected from observational studies. Staple line malformations may lead to a spiral or non-cylindrical gastric tube shape, resulting in higher intraluminal pressure predisposing to gastric leak. Moreover, staple line points of mechanical failure are often associated with areas in increased tissue ischemia, leading to the prompt development of acute leak [48,49,50,51]. This issue can arise due to various factors, such as improper staple line formation, inadequate tissue approximation, or technical errors during the stapling process. Factors like tissue thickness, staple cartridge selection, and staple line tension can also contribute to the occurrence of malformations [48,49,50,51]. When assessing stapling problems, a majority of these were either due to stapler failure to fire or to stapler misfire [48]. When a staple line malformation is identified intraoperatively, immediate intervention is paramount (Figure 1). Surgeons must first assess the extent of the malformation and determine if it compromises the integrity of the sleeve itself or poses a risk of postoperative complications, such as leakage or bleeding. Much research on the area of postoperative complications is further confounded by the use or lack of staple line reinforcement [48]. However, there is clear evidence that staple line malformation does lead to increased morbidity when it goes unnoticed or untreated [50]. Intraoperatively, several strategies can be employed to address staple line malformation. These include reinforcing the staple line with additional sutures or staples, oversewing the affected area, or applying tissue sealants to enhance hemostasis and seal potential leaks [48,49]. Most commonly, suturing over the affected area or attempting to refire a stapler to exclude and excise this compromised area allows for correction of the malformation. As described by Clapp et al., most surgical stapler companies do extensive research and modification of their devices based on these intraoperative issues [48]. While there is likely underreporting of these events, they do remain a rare complication. However, most metabolic and bariatric surgeons are well versed in how to handle these when they do occur [31,48].

## 6. Stapling the Orogastric Tube or the Temperature Probe

**Figure 2:**
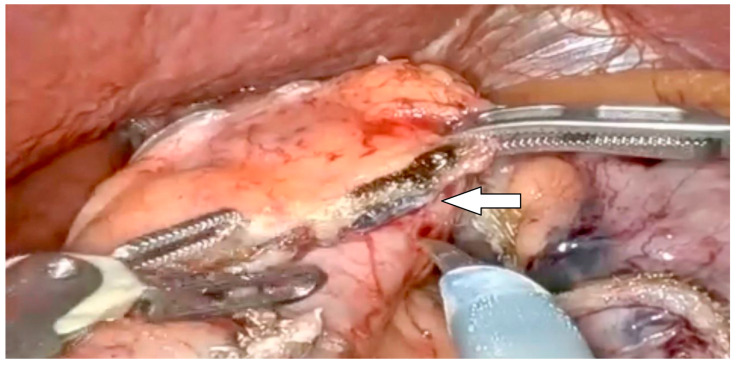
Inadvertent stapling of the orogastric tube.

During sleeve gastrectomy surgery, complications arising from the inadvertent stapling of the orogastric tube (OGT), bougie, or temperature probe can pose significant challenges. The OGT is the most commonly stapled intraluminal device followed by temperature probes and then bougie devices [52,53] (Figure 2). Such mishaps typically occur due to improper positioning or inadvertent incorporation of these instruments within the tissue being stapled due to lack of knowledge of these being present at time of stapling. When this complication arises, immediate recognition and appropriate action are crucial. If stapling occurs, surgeons should cease firing the stapler immediately to prevent further damage. Additionally, they should assess the extent of damage caused by the stapling and evaluate if any vital structures are compromised [52,53]. Intraoperatively, the immediate course of action involves carefully dissecting and releasing the instrument from the stapler, ensuring no residual damage is inflicted [53]. A case series of inadvertent stapling of the orogastric tube was described by Çalıkoglu, reporting three cases of iatrogenic stapling of the orogastric tube (OGT). In these cases, the stapling was immediately stopped until full retrieval of the tube was reached in order to avoid double entrapment [53].

Techniques such as gentle traction and the use of dissecting instruments under direct visualization may aid in the safe removal of the stapled instrument without causing additional harm.

Furthermore, once the instrument is dislodged from the stapler, meticulous inspection of the surrounding tissue should be conducted to identify any potential injuries or bleeding that may have resulted. Hemostasis should be promptly achieved if necessary. In most cases, the resultant defect in the staple line should be oversewn to prevent potential leaks postoperatively. If these are not identified intraoperatively, usually they are promptly recognized postoperatively while attempting to remove these devices. If this occurs, most commonly the patient will require reoperation to surgically correct this error, however, there are some instances where this can be managed endoscopically as well [54]. Overall, stapling devices is uncommon however can cause significant morbidity if not immediately recognized [53,54]. In most instances, this can be managed by removal of the device and then oversewing or restapling of the staple line in question [53,54].

Given the seriousness of these complications, several centers have developed protocols to prevent these events from happening. One method described by Abu-Gazala et al. included a secondary timeout prior to stapling to confirm the removal of all unnecessary tubes per os. While the bougie may stay in place, the removal of the OGT, temperature probes, and any other device prior to stapling helps reduce the incidence of these events. Having close communication with the anesthesia staff during the stapling period has been shown to help avoid this event [53]. In conclusion, in order to minimize these errors, the implementations of a special preoperative protocol including a checklist and effective communication and cooperation between professional personnel (i.e., surgeon, anesthesiologist, and nursing staff) is paramount.

## 7. Phytobezoar Formation

Phytobezoars are commonly defined as fiber-rich residues of vegetables and fruits [55]. The most commonly reported complications associated with phytobezoars are gastrointestinal tract ulceration and obstruction [56]. Very few cases of phytobezoar following SG were reported in literature, but, as the number of SG performed globally is increasing, more cases are expected to be found in the future [55,56,57,58,59]. The formation of phytobezoars after SG can be attributed to gastric stenosis, especially at incisura angularis; altered gastric motility; the low acidity of the gastric environment under mastication; and altered pyloric function [57]. The most commonly reported symptoms and manifestations of phytobezoar formation are the following: vague abdominal pain, bloating, nausea and vomiting, early satiety, dysphagia, anorexia, weight loss, gastrointestinal hemorrhage, and constipation. Nevertheless, the above-mentioned clinical presentations can be misdiagnosed as adhesive small bowel obstruction. The diagnosis can be suggested by an upper gastrointestinal fluoroscopy or abdominal CT scan and further confirmed with endoscopy [57]. Treatment options include chemical enzyme therapy with papain, endoscopic fragmentation, and removal. Surgical treatment should be reserved for cases with persistent symptoms, non-responding to less invasive options. A case of a large gastric phytobezoar in the body of the stomach associated with antrum rotation was described by Aryannezhad et al. In this case, the phytobezoar underwent endoscopic fragmentation and removal using a snare; later, a conversion of LSG to Roux-en-Y gastric bypass (RYGB) was performed [55]. Phytobezoar formation following SG can be prevented by several intra and postoperative strategies, such as avoidance of gastric stricture at the incisura angularis, helical twist of the sleeved stomach, adequate nutritional counseling, long-term medical and nutritional follow-up, and eating habit assessment. Moreover, bariatric patients require proper postoperative dietary counseling by a multidisciplinary team in order to be instructed regarding the importance of adequate eating habits (i.e., consumption of small meals, increased fluid intake, avoidance of foods with high-fiber content, and adequate oral hygiene and food chewing) [55,56].

## 8. Pancreatic Leak and Fistula

Pancreatic leak after SG has been scarcely reported in the literature; nevertheless, it can be a potentially lethal complication and especially difficult in the abdomen mainly due to pancreatic trauma or injury [60,61].

Clinical presentation of a pancreatic leak can vary; patients usually show fever, tachycardia, high white blood cell count, and abdominal pain. The initial diagnosis is usually made by abdominal CT scan with IV contrast showing a left upper quadrant collection with no evidence of air or contrast extravasation from the dissected stomach [60]. The definite diagnosis is reached by detection of increased lipase and amylase levels in the drained fluid. Management of pancreatic leaks depends on patient status at diagnosis. In the case of a stable patient with no response to resuscitation efforts, a percutaneous drainage can be attempted. However, in the case of a patient with hemodynamic instability, laparoscopic exploration should be performed on an urgent basis with further washout with drain placement to monitor the total output. In conclusion, pancreatic leak is an underreported, but dreadful complication after SG, especially in patients with previous complex abdominal surgery causing severe adhesions between the pancreas and posterior stomach [60].

## 9. Gastro-Colonic (GC) Fistula

Gastro-colonic (GC) fistula following primary laparoscopic sleeve gastrectomy has been scarcely reported in the literature. The first case series was reported in 2015 by Nguyen et al. describing one case of gastro-colonic fistula occurring 6 months after primary SG treated with the execution of an open esophagojejunostomy and subtotal colectomy with an ileum to descending colon anastomosis [62]. Since then, other cases of gastro-colonic fistula after primary SG have been reported in literature. The mainstay of therapeutic intervention is determined by the duration from primary surgery. In case of acute GC fistula occurrence (i.e., presentation within 30-postoperative days), minimally invasive treatment with endoscopic or laparoscopic suturing with omental patch may represent a feasible and safe option. Nevertheless, cases of chronic GC (i.e., failure of nonoperative treatment beyond 12 weeks) rarely respond to conservative treatment, and they often require definitive operative management [63]. The main mechanism behind the formation of GC fistula relies on the chronicization of the gastric leak and the subsequent creation of a fistulous tract between the stomach lumen and colon or staple line erosion due to leaked gastric content [62].

Clinical presentation of GC is not specific. Patients can usually present to the emergency department or outpatient clinic with acute onset abdominal pain radiating to the left shoulder and a fever, malnourishment, and recurring episodes of syncope, hypotension, and tachycardia, less often with coffee ground or feculent vomiting, melena, inability to tolerate food and malnutrition. The final diagnosis is usually made by upper endoscopy and barium swallow or abdominal CT scan with medium contrast per os showing a fistulous tract between the stomach and colon [62].

According to the most updated literature, early cases of GC fistula were successfully treated with endoscopic management using over-the-scope clip closure of the fistula opening on the stomach side and a heme clip at the opening of the fistula at the colonic side with distal gastric stenosis balloon dilatation or with endoscopic internal drainage (EID) by inserting double pigtail stents (DPS) [64,65,66]. In the remaining cases of chronic GC fistula, surgical intervention was attempted after nonoperative treatment failure. In 2 cases, a laparoscopic resection of the GC fistula was performed with or without omental interposition [67,68,69]; in the other cases, a definitive open or laparoscopic esophago-jejunostomy with total or partial gastrectomy with Roux-en-Y reconstruction was performed [63,70]. Only one case of a salvage robotic Roux-en-Y fistulojejunostomy was reported as a possible surgical option for chronic GC fistula after SG [71]. The two most recent cases of chronic GC fistula after SG were reported by Shin et al. and Badaoui et al., where the patients were treated by laparoscopic takedown of the GC fistulae associated with a Roux-en-Y fistulojejunostomy and by laparoscopic conversion to Roux-en-Y gastric bypass, respectively [72,73]. In the long term, patients treated for gastro-colic fistula should be referred and followed up by an expert bariatric surgeon with a multidisciplinary team in a specialized center in order to avoid any risk of malnutrition, and proper nutritional and endoscopic assessments should be performed over time.

## 10. Gastro-Pleural (GP) and Gastro-Bronchial (GB) Fistula

The pathogenesis of gastric fistula relies on the theory of vascular necrosis since devascularization during gastric stapling causes ischemia in the gastric suture line, leading to necrosis and the development of a leak that if unmanaged may lead to a fistulous tract and the subsequent formation of an inflammatory phlegmon [74,75]. The inflammatory phlegmon can eventually erode through the diaphragm, setting up an inflammatory process resulting in a pathologic communication between the stomach the bronchial tree or the pleura causing a gastro-bronchial (GBF) or gastro-pleural fistula (GPF), respectively [76,77]. The persistence and evolution of untreated leaks into chronic fistulas are related to the increased intraluminal pressure in the newly sized stomach, even if no strictures at the incisura angularis were created [76].

The first case of GPF was reported in 1960 by Markowitz and Herter as a communication between the stomach lumen and the pleural space following esophageal surgery. Since then, many other cases have been reported in literature, and SG has been identified as one of the main causes of GPF formation [62,78]. GBF development, on the other hand, is mostly related to the spillage of the gastric acid content following a gastric leak with the formation of a subphrenic abscess and continued spreading to above the diaphragm or by directly eroding it. The main consequence of the diaphragm is the formation of a lung abscess, which may lead to communication with the bronchial tree [78]. The accurate incidence of both GBF and GPF is still underreported. According to the work of Silva et al., the mean period of occurrence of GBF after SG was 7.2 months, and GPF has been reported to present as early as three months or as late as 13 years post-procedure [62,79].

The clinical presentation of GBF and GPF is often deceptive with patients being either clinically stable or unstable shortly after SG. The most commonly reported symptoms are fever, dyspnea, productive cough upon swallowing, recurrent respiratory infections, abdominal pain, and hemoptysis [79,80,81]. However, in these cases, other differential diagnoses should be excluded, such as pulmonary embolism, pleural effusions, and atelectasis [82,83,84,85,86].

**Figure 3:**
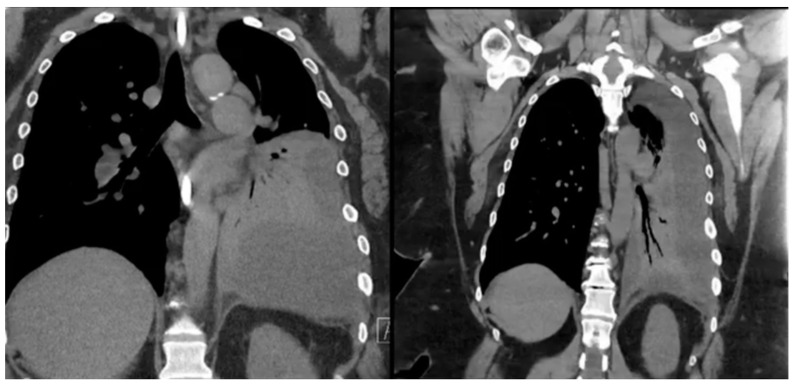
Chest computed tomography (CT) scan showing obliteration of the left lung space in a patient with gastro-pleural (GP) fistula.

Definitive diagnosis of GBF or GPF is usually made with imaging methods. Diagnostic workup includes a wide variety of imaging modalities showing an aero-digestive communication, such as contrast-enhanced CT, barium swallow, Gastrografin study, or an upper GI series. In some cases, methylene blue swallow can be also used in order to assess the presence of blue dye in the chest tube or percutaneous drainage, aiding in the diagnosis of fistulization (Figure 3). Despite the scarcity of data (due to the rarity of this complication) on the gold standard diagnostic tool, a contrast study of the upper gastrointestinal tract is the widely accepted means of diagnosing these two rare complications after SG [80]. Upper endoscopy itself cannot diagnose GBF or GPF, but it can help identify the fistula origin, define the anatomy, and minimize the need for an invasive approach. However, if coupled with fluoroscopy, EGD becomes the method of choice for diagnosis. Bronchoscopy might be helpful but does not always manage to identify the bronchial fistula orifice even after oral methylene blue administration [79,80,81].

Once a diagnosis is made, the treatment should be tailored to the patient’s clinical status. In the absence of major signs of hemodynamic instability and sepsis, an initial nonoperative approach should be attempted [87,88,89,90]. Initial treatment with bowel rest and intravenous broad-spectrum antibiotics is paramount in addressing the concomitant lung infection. In cases of antibiotic therapy failure, CT-guided percutaneous aspiration or drainage could be an option [85]. In recent years, with the increase in bariatric procedures and advancement in endoscopic techniques, the endoscopic approach has become a milestone in the management of GBF and GPF after SG. The use of self-expandable metallic stents (SEMS) has gained popularity since it has been reported to be safe and time-saving, with a procedure-reported mortality of around 2.2% [89]. Other endoscopic interventions include fibrin glue application, endo-suturing, clipping, and balloon dilatation [90,91,92,93].

In some cases, the above mentioned procedures are unsuccessful, and definitive surgical management is required. Silva et al. reported the following surgical options for GBF after SG: a laparoscopic approach with conversion to RYGB or open thoracoabdominal access with left lower lobe resection, diaphragm debridement, completion gastrectomy and creation of Roux-en-Y esophagojejunostomy. In the case of diaphragm erosion with lung involvement, a total gastrectomy with esophagojejunostomy, lung resection with diaphragm resection, and associated reconstruction could be an option [80]. To note, surgical treatment has higher rates of postoperative complications [81]. According to a recent work by Ghanem et al., a laparo-endoscopic approach has been employed for the treatment of recalcitrant chronic GPF, performing a laparoscopic adhesiolysis combined with endoscopic exact identification of the fistulous ostium and concomitant placement of BioGore A^®^ fistula plug and gastric stent [94] following a complete fistula healing with no reported postoperative complications in the long term.

In conclusion, GBF and GPF are rare complications after LSG that are associated with high mortality when left untreated. The management of these rare complications is best approached by a multidisciplinary team and a step-up approach; advanced endoscopy may play a pivotal role in these cases. First, given the patient’s hemodynamic stability, starting with a conservative approach with noninvasive measures is suggested to be followed by minimally invasive options and finally, if the above options fail, definite surgical procedures.

## 11. Portomesenteric Venous Thrombosis (PMVT)

Portomesenteric venous thrombosis (PMVT) can be defined as partial or complete occlusion of the portal and/or mesenteric veins. Its incidence following SG has been reported to be between 0.3 and 1% [95]. According to the most recent literature, PMVT can occur within 22.4 ± 216.5 days, with most cases reported in the first month after surgery [96].

Several risk factors have been described to contribute to PMVT after LSG. They can be divided in the following categories:

- Independent risk factors: These factors include smoking, oral contraceptive use, genetic predisposing conditions for thrombophilia, male sex, baseline BMI, previous history of VTE, history of cancer [97].
- Intraoperative or surgical-procedure related risk factors: Prolongedprolonged liver retraction may cause liver congestion and clot formation. The mechanical or thermal effect when ligating the right gastroepiploic and short gastric vessels may cause reflux in close proximity to the splenic vein, promoting thrombus formation. Moreover, CO_2_ insufflation with hypercapnia-induced vasoconstriction, reverse Trendelenburg position, and increased intraabdominal pressure, especially above 14 mmHg, may predispose to vasospasm and decrease portal blood flow, leading to thrombosis. Eventually, the endothelial damage-induced inflammatory response, especially during the manipulation of the splenic vasculature and pancreatic tissue while opening the lesser sac, can enhance t thrombus formation and alter the coagulation pathway [96,98,99,100].
- Postoperative factors: Dehydration due to a reduced gastric capacity and hypovolemia can promote thrombus formation. For this reason, avoiding dehydration and exposure to heat for the first postoperative month is recommended [101,102].

The clinical presentation of PMVT can be vague, with symptoms potentially mimicking other common medical conditions. The most commonly reported symptoms are abdominal pain, nausea with or without vomiting with an overall incidence rate of 91.8% and 30.8%, respectively, according to a recent meta-analysis. Other uncommon symptoms are fever, hematemesis, and rectal bleeding, which is reported in less than 10% of cases [96,100].

The gold standard diagnostic method for PMVT is contrast-enhanced CT scan with portal venous phase since it has been widely acknowledged to have the highest sensitivity compared to Doppler ultrasound in the detection of splanchnic vein thrombi, particularly in the splenic and superior mesenteric veins; nevertheless, it is not operator dependent [96].

The optimal management of PMVT after LSG has not been clearly elucidated yet. All therapeutic strategies aim at two main goals: prevention of PMVT sequela by halting the thrombus formation and treatment of acute complications of PMVT (i.e., intestinal infarction with necrosis). In the absence of proper treatment, the overall mortality of PMVT has been reported to be up to 20–50% [100]. According to a recent systematic review and meta-analysis from Giannis et al., most cases of PMVT after SG (93.4%) with no signs of intestinal ischemia are hospitalized and treated with therapeutic anticoagulation consisting of unfractionated heparin (UFH), vitamin K antagonists (VKA), or low-molecular-weight heparin (LMWH). The optimal duration of treatment with systemic anticoagulation has not been definitively established. Some papers recommend 3–6 months of anticoagulation, while others suggest a longer duration of anticoagulation therapy, ranging from 6–12 months [96,98]. Measuring anti-factor Xa levels has been suggested to be an option for enoxaparin dose adjustment since it has been reported in literature that the majority of critically ill and obese patients receive inadequate dosing of enoxaparin for DVT prophylaxis [103]. Nevertheless, routine measurement of anti-factor Xa levels should be done until the target weight-adjusted enoxaparin dosing is achieved [104].

In the case of failure of therapeutic anticoagulation, in the absence of intestinal necrosis, the use of thrombolytic therapy or surgical embolectomy has been reported in 4.4% and 2.2% of cases, respectively [96]. Unfortunately, when there is evidence of peritonitis or bowel wall ischemia and a lack of response to the above-mentioned therapeutic options, surgical intervention with bowel resection is mandatory. In a recent meta-analysis, the need for bowel resection and splenectomy in the setting of PMVT was reported to be 10.6% and 1.1%, respectively [96].

In literature, several cases of successful PMVT management are reported. According to the work of Karaman et al., a case of a 35-year-old male who developed PMVT 15 days after LSG was described. He underwent abdominal CT scan with IV contrast showing a thrombus elongating from the superior mesenteric vein to the portal vein, causing necrosis in a 40-cm small-bowel segment that required emergent laparotomy and resection with anastomosis of the interested tract. Eventually, the patient was discharged on postoperative day 7 without any further complications [99]. In the same work cited above, in 75 patients (72.1%), PMVT was successfully managed with anticoagulation therapy only. However, in 27 cases (25.9%), surgical intervention (i.e., bowel resection ± anastomosis, and/or thrombectomy) was further required following failure of conservative treatment [99].

Regarding the prevention and prophylaxis of PMVT after SG, evidence is still lacking. Based on the most updated literature and most recent findings, recommended protocols of PMVT prophylaxis range from intermittent pneumatic compression with early mobilization alone to the addition of chemoprophylaxis [101,104,105]. The use low-molecular-weight heparin for postoperative chemoprophylaxis should be recommended for all patients since it has been shown to be more effective compared to unfractionated heparin and associated with a reduced risk for bleeding [106]. Nevertheless, the duration and dosage of postoperative chemoprophylaxis is still unclear; to address this issue, since most cases of post-LSG PMVT occurs within 30 days of discharge, it should be recommended to extend the duration of prophylaxis up to four weeks after the operation [107]. Other authors suggested the use of a VTE risk calculator to stratify patients and determine patient-specific dosing and duration of postoperative chemoprophylaxis [104,108].

In conclusion, PMVT is a rare but potentially fatal complication after SG, with complex management requiring a multidisciplinary team. Several prothrombotic risk factors have been identified that require an adequate preoperative assessment, including a thrombophilia workup test. Since most post-surgery VTE occurs within 30 days of discharge, we strongly recommend a duration of postoperative thromboprophylaxis with LMWH of at least 4 weeks for higher risk patients.

## Data Availability

Not applicable.
